# SparsePool: A Graph Pooling Framework via Sparse Representation for Graph Classification

**DOI:** 10.3390/s26092627

**Published:** 2026-04-23

**Authors:** Zehan Li, Xuemeng Zhai, Hangyu Hu, Jiandong Liang, Guangmin Hu

**Affiliations:** 1 School of Resources and Environment, University of Electronic Science and Technology of China, Chengdu 611731, China; lizehan@std.uestc.edu.cn (Z.L.);; 2 School of Information and Communication Engineering, University of Electronic Science and Technology of China, Chengdu 611731, China

**Keywords:** graph neural networks, quantum computing, graph classification, sparse representation

## Abstract

Graph neural networks (GNNs) have achieved great success in graph classification, with graph pooling methods being widely adopted for related tasks. Existing approaches typically rely on node ranking or clustering to coarsen graphs, but often fail to effectively leverage global structural information, leading to loss of critical substructures and limited interpretability—key limitations in molecular analysis and social network mining. To address these issues, we propose SparsePool, a graph pooling method that integrates node features and structural patterns through atomic decomposition. By dynamically decomposing graphs into interpretable atomic units via Boolean matrix factorization, SparsePool preserves semantically meaningful substructures while providing transparent evidence of retained patterns. We further introduce an Atomic Pooling Neural Network (APNN) for graph representation learning. Extensive experiments on relevant benchmarks including biochemical and social network datasets demonstrate that SparsePool outperforms state-of-the-art pooling methods, achieving an average classification accuracy improvement of 1.03% over baseline models while reducing structural information loss. We also discuss its compatibility with emerging quantum computing paradigms, such as quantum-accelerated sparse decomposition, as a promising direction for scaling graph processing in industrial contexts.

## 1. Introduction

In critical industrial sectors—ranging from drug molecule discovery in healthcare to supply chain optimization and social network analysis—graph neural networks (GNNs) have become pivotal tools, owing to their powerful capacity for modeling complex relational data [1,2,3,4]. GNNs have achieved remarkable success in many graph-level tasks, such as graph classification [5], graph regression [6], and graph generation [7]. However, when deploying GNNs in safety-critical and high-risk industrial environments, model interpretability, decision transparency, and faithful preservation of the underlying structure are requirements as crucial as predictive performance. Unlike node-level tasks that mainly use graph convolutional networks (GCNs) [8], graph-level tasks require holistic, compact representations for input graphs with varying sizes and topologies. Therefore, pooling serves as a fundamental component to compress node representations into an effective global representation.

Existing pooling methods, apart from the simplest global pooling that averages all nodes, include node selection-based or node clustering-based approaches to aggregate node information [9]. However, despite the considerable achievements of these methods in various tasks, many problems remain. First, node selection-based pooling methods such as TopKPool [10], SAGPool [11], and ASAP [12] cut unnecessary nodes based on designed ranking strategies in each pooling layer, but this approach leads to information loss. Although other node clustering-based methods avoid this problem, artificially specified node compression methods still cannot prevent damage to local graph structures [9]. Furthermore, these pooling layers do not explicitly incorporate topological information when calculating node clusters, which may result in performance degradation.

To address these challenges, we propose SparsePool, a novel graph pooling method that achieves interpretability through sparse representation and atomic decomposition. Unlike traditional strategies reliant on ranking or sampling, SparsePool dynamically learns a set of semantically meaningful “atoms” from the graph via Boolean matrix factorization and uses these atoms as super-nodes to construct the pooling assignment matrix. The core of this approach lies in sparse coding, which represents each node as a linear combination of a few critical primitives from an atomic dictionary, thereby explicitly capturing and preserving the discriminative key substructures within the graph. This process inherently ensures the fidelity of the global structure, and because the resulting atoms carry intuitive semantics (e.g., functional groups in molecules or community motifs in social networks), it naturally provides interpretability for pooling decisions—i.e., the model can explicitly indicate which structural patterns are preserved and why. Thus, SparsePool not only enhances downstream task performance but also meets the urgent demand for structural transparency and interpretability in analyzing complex industrial graph data. It is worth noting that the sparse representation and matrix factorization framework underlying this method exhibits algorithmic affinity with quantum computing paradigms, which could inspire future explorations of classical–quantum hybrid systems for efficient and interpretable graph processing, as briefly discussed in Section 5.

Experiments show that our method improves the average graph classification accuracy in multiple benchmark datasets, including bioinformatics and social network datasets, confirming its effectiveness. In complex scenarios, visualization results show that these atoms correspond to biological functional modules. Notably, this method can be used as a plug-and-play module compatible with mainstream GNN architectures (such as GCN [8] and GAT [13]), providing a collaborative optimization solution for the structural sensitivity and interpretability of graph pooling techniques. The main innovations of this paper are as follows:

- We propose SparsePool, a novel and interpretable graph pooling method for constructing assignment matrices. Built on sparse representation principles, SparsePool dynamically decomposes input graphs into interpretable atomic units, enabling seamless integration with existing mainstream GNN architectures.
- We introduce a decomposition mechanism constrained by the global information bottleneck—a principle that forces the network to compress data while retaining maximally predictive structural patterns. This explicit mapping between derived atoms and the original topology ensures high fidelity to the graph’s structural properties and addresses the suboptimal substructure problem found in traditional pooling methods.
  Specifically, the decomposition is achieved through the synergistic effect of sparse representation decomposition and the constraints of the global information bottleneck, which guides the retention of structurally critical information during decomposition. Compared with traditional pooling methods, SparsePool establishes an explicit mapping between atoms and the original graph structure.
- Experimental results show that SparsePool outperforms state-of-the-art graph representation learning methods in graph classification tasks, validating its superiority.

## 2. Related Work

### 2.1. Graph Pooling

Unlike node-level tasks that mainly use graph convolutional networks (GCNs) [8], graph-level tasks require holistic representations of graph structure inputs. Therefore, for graph-level tasks, the pooling mechanism is a crucial component that compresses the node representations generated by graph neural networks into smaller graphs or single vectors. To obtain effective and reasonable graph representations, many graph pooling designs have been proposed, roughly divided into flat pooling [14] and hierarchical pooling. The former directly generates graph-level representations in one step, mainly using the average or sum of all node embeddings as the graph representation (such as [5,14,15,16,17,18]), but these flat-pooling methods lead to the loss of key substructure information and cannot effectively capture the hierarchical semantics of the graph. In contrast, the latter gradually coarsens the graph into smaller graphs through node clustering or node selection [19].

The node selection pooling methods (such as [20,21,22,23,24,25,26,27,28]) select important nodes through learnable projection vectors. Although these methods are parameter-efficient and more suitable for large-scale graphs, they ignore local topological correlations due to relying solely on global feature ranking for node selection, leading to irreversible loss of hierarchical substructures, such as protein functional domains and social network community cliques.

Therefore, another design based on node clustering was proposed to avoid these problems. Specifically, node clustering groups nodes into groups to form coarsened graphs (such as [29,30,31]), where the nodes of the original graph are merged into a set of clusters. Although this mitigates information loss, disruption of local graph structures still exists due to fixed node compression ratios.

To address the limitation that existing methods neglect the relationships between substructures formed by node assignments, StructPool [32] addresses this limitation by transforming the learning of assignment matrices into a structured prediction problem. It employs conditional random fields to capture structural information between nodes and designs functions to incorporate graph topological information. MinCutPool [33] uses multilayer perceptrons to learn node assignment matrices. MemPool [34] proposes a graph coarsening memory layer for joint graph representation learning; it leverages multiple sets of keys and convolution operations in this memory layer to construct assignment matrices, thereby avoiding dependence on inter-node structural information. WGDPool [35] additionally embeds the weight information of edges as input to the graph. In a parallel line of research, weighted graph clustering methods such as [36] have explored graph contraction and attention to edge weight to reduce the graph scale while preserving important nodes and suppressing noisy connections. Although these methods target clustering rather than hierarchical pooling, they share with SparsePool the core idea of leveraging structural information for compact graph coarsening.

Despite these efforts, existing methods still suffer from mutual interference among substructures during formation—a suboptimality problem that persists because node assignment matrices are not guided by globally coherent structural patterns. Consequently, damage to graph structures remains unavoidable.

### 2.2. Network Sparse Representation

Sparse representation algorithms, first proposed by Olshausen [37], are tools for analyzing non-stationary signals. With continuous development, they have been widely applied in image processing, fault diagnosis, compressive sensing, face recognition, machine learning, and medical imaging (such as [38,39,40,41]). The core idea is to represent initial input signals as linear combinations of a small number of basic signals (atoms) from a dictionary. Sparse representation methods exhibit excellent performance and good interpretability.

As research on complex networks matures, numerous models have been proposed, including community structures, random models, link prediction, and high-order network structures. Recently, sparse representation has been extended to complex network analysis: the sparse representation method for complex networks considers the inherent sparsity of large-scale complex networks, treating networks as signals and applying sparse representation to network analysis [42]. Based on dictionary learning, this method models networks, learns dictionary matrices of the original networks, and extracts atoms as the basic structures of networks. These atoms, similar to motifs, are high-order network substructures. Compared with motifs, this method does not require predefined sizes and can extract multi-node high-order substructures from networks.

Sparse representation of complex networks can be used for network decomposition, compression, and dimensionality reduction. By sparsely decomposing networks to mine the basic structural patterns that compose the original networks, it provides network researchers with tools for network structure analysis and insights into tasks such as network classification and structural analysis in complex networks.

## 3. Method

In this work, we propose a graph pooling method based on sparse representation and atomic decomposition. By applying sparse representation on the graph data, this method extracts atomic structures with global semantics from the graph to guide clustering of nodes and the construction of node assignment matrices. Based on our SparsePool method, we propose an atomic pooling-based neural network for graph representation learning.

### 3.1. Notations and Problem Statement

#### 3.1.1. Graph Notations

In this paper, we adopt standard graph notation to formalize the graph-structured data. A graph is denoted as G=(V,E,X), where $V=\{v_{1},v_{2},\ldots ,v_{N}\}$ represents the set of nodes with N=|V| being the number of nodes, and E⊆V×V denotes the set of edges. The node attribute matrix is denoted as $X\in R^{N\times d}$, where $X_{i}\in R^{d}$ corresponds to the attribute vector of node $v_{i}$ with *d* being the dimension of node features. The structure of the graph is represented by an adjacency matrix $A\in {\left\{0,1\right\}}^{N\times N}$, where $A_{ij}=1$ if $(v_{i},v_{j})\in E$ and $A_{ij}=0$ otherwise; for undirected graphs, A is symmetric with $A_{ii}=0$ (no self-loops).

#### 3.1.2. Problem Statement

The graph classification task aims to learn a mapping from graph-structured data to predefined categorical labels. Formally, given a training set $D_{\mathrm{train}}\;=\;{\left\{\left(G_{i},y_{i}\right)\right\}}_{i=1}^{n_{\mathrm{train}}}$ consisting of $n_{\mathrm{train}}$-labeled graphs—where $y_{i}\in Y$ denotes the graph-level label of $G_{i}$ and Y is the label space—the goal is to train a model f:G→Y that can accurately predict the labels of unseen graphs in the test set $D_{\mathrm{test}}={\left\{\left(G_{j},y_{j}\right)\right\}}_{j=1}^{n_{\mathrm{test}}}$. Critical challenges in this task arise from the inherent structural diversity across graphs (e.g., varying sizes, edge densities, and topological patterns) and the need to integrate both node attributes (if available) and relational information encoded in edges to capture discriminative graph-level features.

#### 3.1.3. Graph Pooling

We define graph pooling operations based on the graph structure G=(V,E,X) with |V|=N nodes and node feature matrix $X\in R^{N\times d}$. A pooling function $P:G\mapsto G^{\prime }$ maps the original graph to a coarsened graph $G^{\prime }=\left(V^{\prime },E^{\prime },X^{\prime }\right)$ where $|V^{\prime }|=N^{\prime }\leq N$. The node selection mechanism is characterized by a matrix $S\in R_{\geq 0}^{N^{\prime }\times N}$, where $S_{i,j}$ indicates the contribution of node j∈V to node $i\in V^{\prime }$. The coarsened node features are given by $X^{\prime }=SX$. For edge reconstruction in $G^{\prime }$, the adjacency matrix $A^{\prime }\in R^{N^{\prime }\times N^{\prime }}$ is typically derived from A via $A^{\prime }=SAS^{⊤}$ to preserve structural dependencies. When focusing on graph-level representation learning, the pooling function may reduce *G* to a single vector $h\in R^{d^{\prime }}$ through global aggregation (e.g., h=mean(X) or h=max(X)), where $d^{\prime }$ denotes the dimension of the graph-level embedding.

### 3.2. Graph Sparse Representation and Atomic Extraction

Instead of relying on predefined clustering or node selection heuristics, SparsePool constructs the pooling assignment matrix by first decomposing the graph into a set of interpretable atoms—semantically meaningful substructures—via sparse representation. This process consists of three steps: ego-network sampling, Boolean matrix factorization, and assignment construction. Algorithm 1 details this Boolean matrix factorization procedure.
**Algorithm 1** Boolean Matrix Factorization for Atomic Extraction**Require:** Input matrix $Y\in {\left\{0,1\right\}}^{m\times n}$, confidence threshold ϕ, coverage weight ω, maximum number of atoms $k_{\max}$, reconstruction error tolerance τ
**Ensure:** Dictionary matrix $D\in {\left\{0,1\right\}}^{m\times k}$, sparse code matrix $C\in {\left\{0,1\right\}}^{k\times n}$
1:Initialize residual matrix X←Y, dictionary D←[], sparse code C←[], column count k←02:Compute row correlation matrix *R* using threshold ϕ3:**while** $k<k_{\max}$ and ${∥X∥}_{1}>\tau$ **do**4:    **for** each candidate column $r_{l}$ in *R* **do**5:        Generate row vector $c^{l}$6:        Compute coverage $\mathrm{cover}(Y,D\cup \left\{r_{l}\right\},C\cup \left\{c^{l}\right\},\omega )$7:     **end for**8:     Select column $r_{l^{*}}$ with maximum coverage9:     Append $r_{l^{*}}$ to *D* and $c^{l^{*}}$ to *C*10:   Update X←X−D∘C11:     k←k+112:**end while**13:**return** *D*, *C*


#### 3.2.1. Ego-Network Sampling and Vectorization

Directly applying sparse decomposition to the full adjacency matrix tends to capture only trivial neighbor patterns and overlooks higher-order structural interactions. To obtain a richer set of substructures, we sample ego-centered networks for each node. Specifically, for each node $v_{i}$, we extract its induced subgraph including up to S−1 highest-degree neighbors, forming an ego-network of at most *S* nodes. Each ego-network is represented by its adjacency matrix, which is then vectorized into a column of length $S^{2}$. Stacking all such columns yields the sampling matrix $Y\in {\left\{0,1\right\}}^{S^{2}\times N}$, where each column corresponds to a local structural view around a node.

#### 3.2.2. Boolean Matrix Factorization for Atom Extraction

The sampling matrix Y encodes the presence or absence of local structural patterns. To extract recurring substructures, we factorize (Y into a Boolean dictionary matrix $D\in {\left\{0,1\right\}}^{S^{2}\times k}$ and a sparse code matrix $C\in {\left\{0,1\right\}}^{k\times N}$, such that Y≈D∘C, where ∘ denotes Boolean matrix multiplication. Each column of D corresponds to an atom—a prototypical substructure pattern—and each column of C indicates which atoms appear in a given ego-network. The factorization is performed by a greedy column-selection algorithm that maximizes the coverage of 1-entries in *Y* while enforcing sparsity in C [42]. The resulting dictionary captures global structural primitives (e.g., functional groups in molecules or community motifs in social networks) without requiring domain-specific prior knowledge.

### 3.3. From Atoms to Pooling Assignment Matrix

After extracting the Boolean dictionary matrix $D\in {\left\{0,1\right\}}^{S^{2}\times k}$ and the sparse code matrix $C\in {\left\{0,1\right\}}^{k\times T}$, we construct the pooling assignment matrix $S\in R^{k\times N}$. Here, T=N is the number of ego-networks (one per node), *k* is the number of atoms, and *N* is the number of nodes in the original graph. Each atom corresponds to a semantic substructure pattern and will serve as a super-node in the coarsened graph.

To formalize the mapping, we first define the node-ego-network affiliation matrix $M\in {\left\{0,1\right\}}^{N\times T}$, where $M_{j,t}=1$ if node $v_{j}$ belongs to the ego-network centered at node *t* (including the center node itself), and 0 otherwise. By construction, $M_{t,t}=1$ for all *t*. The sparse code matrix *C* encodes which atoms are present in each ego-network: $C_{i,t}=1$ if atom *i* is present in the ego-network centered at node *t*, and 0 otherwise.

The product $CM^{⊤}$ yields a matrix whose (i,j)-th entry counts the number of ego-networks that contain both node $v_{j}$ and atom *i*:

$${\left(CM^{⊤}\right)}_{i,j}=\sum_{t=1}^{T}C_{i,t}\cdot M_{j,t}.$$

Intuitively, if a node frequently co-occurs with an atom across multiple ego-networks, that node should be strongly assigned to that atom. Therefore, we define the assignment weight from node $v_{j}$ to atom *i* as the normalized co-occurrence count:

$$S_{ij}=\frac{{\left(CM^{⊤}\right)}_{i,j}}{\sum_{t=1}^{T}M_{j,t}}=\frac{\sum_{t:v_{j}\in E_{t}}C_{i,t}}{\left|E(v_{j})\right|},$$

where $E_{t}$ denotes the ego-network centered at node *t*, and $E(v_{j})$ is the set of ego-networks containing node $v_{j}$. The denominator normalizes by the number of ego-networks that include node $v_{j}$, ensuring that each column of *S* sums to 1 (i.e., *S* is column-stochastic). This property guarantees that the total assignment mass of each node is preserved across atoms.

The resulting assignment matrix $S\in R^{k\times N}$ directly enables graph coarsening: the super-node features are computed as $X^{\prime }=SX$, and the coarsened adjacency matrix as $A^{\prime }=SAS^{⊤}$. Thus, each super-node aggregates information from original nodes according to their semantic affinity to the corresponding atomic substructure.

### 3.4. Graph Pooling Network Based on Sparse Representation

**Figure 1:**
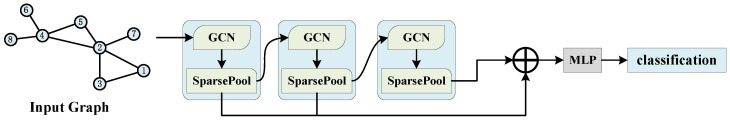
Overview of SparsePool architecture introduces the hierarchical graph classification architecture. Each layer consist of two parts: one graph convolution module (GCN) and one pooling module (SparsePool).

This section introduces the key mechanisms of SparsePool, emphasizing its effectiveness in node selection and information aggregation. Figure 1 illustrates that SparsePool comprises three core modules: graph convolution, atom-guided graph pooling, and the optimization objective. Each module is elaborated on in detail below.

In SparsePool, the core of constructing the node assignment matrix lies in using the learned atoms as semantic super-nodes and directly defining their attribution weights using the atomic activation coefficients of nodes. Specifically, each node of the input graph is first represented as a linear combination of a few significant atoms in the over-complete atomic dictionary through sparse coding, obtaining the sparse activation coefficient vector of the node for the atoms; this coefficient naturally quantifies the semantic correlation strength between the node and the atoms (for example, an oxygen atom in a molecule has a high activation value for the ‘carboxyl group’ atom). Each column of the assignment matrix S corresponds to an atom (super-node), and its elements are filled by the activation coefficients of all nodes for this atom. After normalization, they form the soft assignment weights from nodes to super-nodes. This mechanism ensures that only nodes strongly related to the atomic structure are aggregated with high weights, whereas irrelevant nodes are sparsely suppressed. Finally, through this assignment matrix, a coarse-grained graph is compressed and generated that retains both the semantic meaning of key substructures and the physical interpretability.

Based on SparsePool, we construct a series of networks for graph classification tasks. We first apply a graph embedding layer to generate low-dimensional representations of nodes in the graph, which helps process some datasets with very high-dimensional input feature vectors. Here, we use a GCN layer for node embedding. After the embedding layer, we stack several blocks, each consisting of a GCN layer for advanced feature extraction and a graph pooling layer based on atomic representation for graph coarsening. We feed the output feature matrices of the graph embedding layer and the graph pooling layer based on atomic representation into the classifier.

After obtaining the graph-level representation from the final pooling layer, we apply a two-layer MLP classifier. Specifically, we first aggregate node features using global max pooling, average pooling, and sum pooling, then concatenate the resulting vectors to form a comprehensive graph representation, which is subsequently fed into the MLP for classification.

## 4. Experiments

In this section, we evaluate our method and network on graph classification tasks using biochemical and social network datasets. We further investigate the impact of different graph neural network backbones on model performance and provide interpretability analysis of the learned atomic substructures.

### 4.1. Experimental Setups

#### 4.1.1. Datasets

We use six public datasets to evaluate our method, including two protein datasets, two social network datasets, and two compound datasets from TU Datasets [43]. Meanwhile, for tasks in practical work, a graph classification dataset was constructed using the Twitter social network dataset to distinguish between social networks in the political domain and those in the entertainment domain. Specifically, we manually obtained multi-order egocentric networks of active users related to the political and entertainment domains and constrained their sizes via degree. These experiments, through datasets from different domains, demonstrates that our method can function under various scenarios. The statistical characteristics of the datasets are presented in Table 1.

#### 4.1.2. Models

To validate the superiority of our method, we employed several baseline models, including hierarchical graph pooling methods: DiffPool [29], SAGPool [11], MinCutPool [44], and GMT [45]; node dropout pooling methods: gPool [10], ASAP [12], andTAPool [21]; as well as structure-based pooling: EdgePool [46]. Additionally, we include two widely used GNN backbones, GCN [8] and GIN [47], which employ global mean pooling (i.e., averaging all node embeddings) to obtain graph-level representations. These baselines cover a broad spectrum of graph classification approaches and provide a comprehensive comparison for SparsePool.

#### 4.1.3. Implementation Details

For all baseline methods and SparsePool, we adopt a consistent backbone architecture to ensure fair comparison. Each model consists of three graph convolutional layers (GCN) with hidden dimension 128 and ReLU activation, followed by a pooling layer (or global pooling for non-hierarchical baselines). The final graph-level representation is passed through a two-layer MLP classifier with hidden dimension 64 and softmax output. Dropout with rate 0.5 is applied after each convolutional layer. For hierarchical pooling methods, including DiffPool, SAGPool, MinCutPool, GMT, gPool, ASAP, TAPool, EdgePool, and our SparsePool, we apply pooling after the first and second GCN layers, resulting in two pooling operations per model. The pooling ratio is set to 0.5 for both layers [45]. For GCN and GIN (non-hierarchical baselines), we apply global mean pooling after the final GCN layer to obtain graph-level representations.

All models are trained using the Adam optimizer with an initial learning rate of 0.001, weight decay of $5\times 10^{-4}$, and a batch size of 64. We use the cross-entropy loss function. Early stopping is applied based on validation accuracy with a patience of 50 epochs, and the maximum number of epochs is set to 500. The reported results are the average of 10 independent runs with different random seeds. For all datasets, we adopt the standard 10-fold cross-validation protocol. For each fold, 80% of the data is used for training, 10% for validation, and 10% for testing. The validation set is used for early stopping and hyperparameter tuning. For the Twitter-real dataset, we use a fixed split of 80% training, 10% validation, and 10% testing due to its specific domain structure.

### 4.2. Experimental Results

Table 2 summarizes the performance of SparsePool and baseline methods on seven benchmark datasets. Based on a thorough analysis of the results, we identify several key observations, which we elaborate on in detail below.

First, SparsePool achieves superior performance over most baseline methods and delivers competitive results compared to the best existing models. Specifically, it significantly outperforms previous state-of-the-art approaches across multiple attributed-graph datasets, with accuracy improvements of 1.32% on D&D, 0.84% on PROTEINS, 1.01% on NCI1, 1.09% on NCI109, 0.69% on COLLAB, and 1.09% on Twitter-real.

Notably, SparsePool consistently surpasses both GCN and GIN across all seven datasets. This advantage stems from the fact that these baseline models do not explicitly incorporate graph structural information during global summarization of node representations. In contrast, strong pooling-based baselines such as EdgePool deliberately integrate global topology and task-relevant features during coarsening to preserve discriminative information.

It is particularly worth emphasizing that, even on the most challenging dataset—Twitter-real, which is characterized by high structural complexity and pronounced inter-class similarity—our atom-based approach still maintains discernible discriminatory power. Moreover, it substantially outperforms all other competing methods by a notable margin, demonstrating its robustness and generalizability in handling complex and noisy graph-structured data.

Moreover, SparsePool consistently outperforms most hierarchical graph pooling methods across six datasets. This strongly validates the effectiveness of our atomic pooling operator in extracting meaningful and discriminative substructures. We argue that by explicitly designing the pooling operator from both node and edge perspectives, SparsePool achieves more efficient utilization of the graph’s structural information. In particular, edges play a role beyond merely connecting nodes—they carry critical structural patterns that are not secondary to nodes. Our atomic pooling mechanism successfully captures these informative substructures, leading to enhanced representational capacity and performance.

We conducted experiments on four bioinformatics datasets. SparsePool achieves significantly stronger performance on PROTEINS and DD compared to NCI1 and NCI109. We attribute this difference to the fact that the NCI1 and NCI109 datasets exhibit relatively simpler and sparser graph structures. Under such conditions, the advantage of sparse structure representation is less pronounced, as even conventional methods can obtain reasonably discriminative representations. In contrast, the more complex and densely structured graphs in PROTEINS and DD allow our method to better leverage its capacity to extract globally informative substructures that are difficult for other models to capture. This capability leads to substantially improved representation learning on these more challenging datasets. Furthermore, SparsePool also delivers competitive performance on the Twitter-real dataset, which contains real-valued node attributes.

Among existing approaches, EdgePool is the most similar to SparsePool. However, SparsePool consistently outperforms EdgePool across all datasets. This advantage stems from SparsePool’s ability to leverage atomic structures—local substructures with clear semantic meaning—to capture richer and more discriminative graph representations. While EdgePool learns cluster representations by aggregating all neighboring nodes, our approach introduces a novel strategy that identifies and retains critical atomic structures by jointly considering both the center node and its informative neighbors. This allows SparsePool to form more semantically cohesive clusters and extract optimal substructures that are essential for complex graph reasoning, substantially enhancing its representational power compared to EdgePool.

### 4.3. Dataset-Dependent Behavior Analysis

SparsePool exhibits the strongest performance on datasets characterized by rich hierarchical structures and semantically meaningful higher-order substructures. In particular, datasets representing proteins (e.g., D&D and PROTEINS) and complex molecules contain well-defined functional domains such as α-helices, β-sheets, and various chemical functional groups. These substructures align naturally with the atomic decomposition mechanism of SparsePool, which explicitly extracts interpretable atomic units from the graph via Boolean matrix factorization. The pooling operation then aggregates nodes according to these domain-level semantics, preserving critical structural patterns that are often lost in conventional pooling methods. Consequently, when the underlying data inherently decomposes into such modular units, SparsePool is able to leverage its inductive bias to capture discriminative features more effectively.

SparsePool also demonstrates notable effectiveness on social network datasets, where it extracts complex and semantically meaningful substructures such as star-shaped patterns representing core–periphery relationships, densely connected clusters corresponding to cohesive communities, and bridging structures that serve as critical intermediaries between different groups. These atomic units go beyond simple community detection, capturing nuanced organizational principles that are often overlooked by conventional pooling approaches. While baseline methods such as EdgePool rely on aggregating neighboring nodes without explicitly modeling higher-order structural patterns, SparsePool’s atomic decomposition enables the discovery of these intricate substructures, leading to more discriminative graph representations. The method maintains competitive performance across all benchmarks, consistently outperforming or matching state-of-the-art approaches, demonstrating its robustness and generalizability across diverse graph types.

### 4.4. SparsePool with Different GNNs

The SparsePool proposed in this paper can be flexibly integrated into various graph neural network architectures. To ensure the fairness of comparative experiments and the broad applicability of the method, we first adopt the most widely used GCN as the basic backbone model for learning node representations, and meanwhile introduce GAT, another mainstream architecture, to explore the impact of different convolution operations on model performance. We conduct performance evaluations of SparsePool integrated with these two convolution operators on five public datasets, and the experimental results are shown in Table 3.

It is found that regardless of the convolutional backbone network used, SparsePool combined with different convolution operations achieves significantly better performance than state-of-the-art graph pooling models such as TAPool and EdgePool. For example, the classification accuracy of SparsePool-GAT is 1.72% higher than that of EdgePool on the COLLAB dataset; in addition, SparsePool-GCN yields an average classification accuracy improvement of 2.39% and 1.06% over TAPool and EdgePool, respectively. The above results fully verify the effectiveness of the SparsePool method and also prove that this method has good general applicability and can be efficiently combined with different GNN convolutional architectures.

### 4.5. Interpretability Analysis

The interpretability of SparsePool stems from its core mechanism—atomic decomposition via sparse representation. Unlike conventional pooling methods that compress nodes into abstract vectors, SparsePool decomposes a graph into a set of recognizable substructure units, termed atoms, and explicitly establishes the affiliation between nodes and these atoms. This design endows the intermediate representations and final predictions with inherent transparency.

First, atoms themselves carry semantic interpretability. Through Boolean matrix factorization, each atom corresponds to a frequently occurring subgraph pattern, such as functional groups. These atoms are learned from data without manual annotation, yet their morphologies align well with domain knowledge, making them intuitively understandable to domain experts.

Second, the pooling process is explicit and traceable. The assignment matrix *S* constructed by SparsePool directly quantifies the association strength between each node and each atom: a node is assigned higher weight to an atom if it frequently co-occurs with that atom in local neighborhoods. Consequently, it is clearly known which original nodes constitute each coarsened super-node and to which super-nodes each original node contributes. This property enables backward tracing: any representation generated in the coarsened graph can be traced along the assignment relations to the specific set of nodes and substructures in the original graph.

Moreover, the prediction decision can be attributed to a small set of key atoms. Because the sparse code matrix *C* activates only a few atoms for each graph, the final graph representation is constrained to be a sparse combination of these atoms. Thus, when the model makes a classification decision, we can directly examine which atoms contribute most and explain the decision accordingly. For example, if a molecule is classified as having anti-tumor activity due to the activation of a “purine ring” atom and a “specific side chain” atom, such an explanation directly corresponds to pharmacological knowledge.

**Figure 2:**
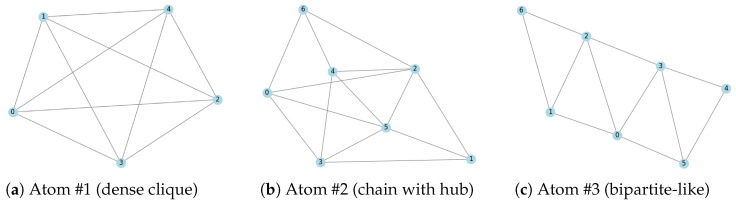
Atomic substructures extracted from the DD protein dataset. Each atom corresponds to a frequently occurring subgraph pattern. Numbers 0–5 denote node indices within the extracted atomic substructure.

To provide concrete experimental evidence, we showcase the atomic subgraphs learned by SparsePool on the DD protein dataset, which contains two classes (Class 1 and Class 2). Figure 2 visualize the subgraph patterns of three representative atoms (reshaped from dictionary columns). Atom #1 exhibits a dense, near-clique structure; Atom #2 shows a chain-like pattern with a central hub; Atom #3 displays a sparse bipartite-like structure. We further compute their average activation proportions (the fraction of nodes in a graph where the atom is activated) across the two classes. The results are as follows: Atom #1 in Figure 2a exhibits similar proportions in both classes (12.3% vs. 10.8%); Atom #2 Figure 2b is activated significantly more frequently in Class 1 (28.4%) than in Class 2 (9.7%); Atom #3 Figure 2c shows the opposite trend, with higher activation in Class 2 (24.1%) than in Class 1 (7.5%). This statistical disparity demonstrates that these atoms capture class-relevant structural information. The above quantitative analysis provides direct and reproducible evidence for the interpretability of SparsePool.

In summary, the interpretability of SparsePool is not achieved through post hoc visualization or attention heatmaps; it is embedded in the pooling mechanism itself. Atoms serve as semantic primitives, the assignment matrix provides a traceable mapping, and sparse coding offers compact evidence for decisions. Together, these elements form a transparent and interpretable framework for graph representation learning.

## 5. Discussion and Future Work

The core computational bottleneck in SparsePool lies in the sparse decomposition procedure, particularly the iterative singular value decomposition (SVD) within the K-SVD algorithm. While the current implementation relies on classical SVD and scales effectively for the benchmark datasets used in this study, the demand for processing ultra-large graph data—such as billion-scale molecular graphs or global supply chain networks—may exceed the capacity of classical methods.

Emerging quantum computing paradigms offer a promising direction to address such scalability challenges. Notably, quantum singular value decomposition (QSVD) and related quantum linear algebra subroutines have been theoretically shown to achieve exponential speedup for large matrix decomposition under certain conditions. In principle, substituting the classical SVD steps in K-SVD with QSVD could enable SparsePool to process industrial-scale graphs with significantly improved efficiency. This would form the basis of a classical–quantum hybrid architecture, where quantum processing units (QPUs) handle computationally intensive decomposition tasks, while classical units manage node aggregation, interpretability analysis, and downstream classification.

We emphasize that the quantum enhancement described above is a future direction rather than a component implemented in this work. The current paper focuses on the design, implementation, and empirical validation of SparsePool as a fully classical method. Nonetheless, the algorithmic compatibility between sparse representation and quantum computation suggests a promising pathway for scaling interpretable graph pooling to real-world industrial applications.

## 6. Conclusions

This paper proposes a novel graph pooling framework based on sparse representation atomic decomposition to address the problems of global semantic structure loss and non-transparent decision-making processes in traditional graph pooling methods. This method decomposes input graphs into interpretable atomic units through dynamic Boolean matrix decomposition, uses data-driven atomic structures to guide node clustering, and optimizes connectivity by combining node degree constraints, achieving precise retention of key substructures (such as biological functional modules and social hubs) and transparent pooling decisions, solving the problem of insufficient expressive power of traditional methods for high-order structures. As a plug-and-play module embedded in mainstream GNNs such as GCN/GAT, it improves graph classification accuracy by an average of 1.1–3.1% on six benchmark datasets of social networks and bioinformatics, with significant advantages especially on large sparse graphs like the Twitter-real dataset and complex semantic graphs like DD. This work provides a new paradigm for the collaborative optimization of structural sensitivity and interpretability in graph pooling technology, offering reliable tools for tasks requiring the retention of key substructures, such as molecular function prediction and social community discovery.

While the current work focuses on the classical implementation of SparsePool, we note that its reliance on iterative singular value decomposition presents a natural opportunity for quantum acceleration. As discussed in Section 5, future work may explore the integration of quantum singular value decomposition (QSVD) to scale SparsePool to billion-scale industrial graph data, laying a foundation for classical–quantum hybrid graph processing architectures.

## Figures and Tables

**Table 1:** Statistics of the network datasets.

Dataset	# of Graph	Avg.# of Nodes	Avg.# of Edges	# of Classes	Node Attributes
D&D	1178	284.32	715.66	2	Node label
Protein	1113	39.06	72.82	2	Node label
NCI1	4110	29.87	32.30	2	Node label
NCI109	4127	29.68	32.13	2	Node label
COLLAB	5000	74.49	2457.78	3	None
IMDB-MULTI	1500	13.00	65.94	3	None
Twitter-real	2000	167.33	693.74	2	Node label

**Table 2:** Classification accuracy (%) of different pooling methods and GNN baselines on test sets. Best results are in bold.

Dataset	D&D	Protein	NCI1	NCI109	COLLAB	IMDB-MULTI	Twitter-Real
GCN	73.83 ± 1.19	69.52 ± 2.86	75.39 ± 2.53	76.15 ± 3.03	79.61 ± 1.59	48.40 ± 3.12	71.23 ± 3.26
GIN	74.24 ± 1.19	67.31 ± 3.38	73.36 ± 2.90	72.23 ± 3.11	77.20 ± 1.51	48.25 ± 2.04	66.88 ± 4.96
DiffPool	77.85 ± 2.38	75.27 ± 4.78	76.53 ± 0.91	71.85 ± 1.93	74.78 ± 2.95	44.13 ± 2.66	67.65 ± 3.19
SAGPool	76.48 ± 1.74	72.46 ± 3.05	70.07 ± 1.25	67.79 ± 1.83	78.92 ± 1.60	46.17 ± 1.10	63.89 ± 2.87
MinCutPool	77.73 ± 2.74	75.30 ± 3.71	75.90 ± 2.05	74.26 ± 3.46	70.71 ± 4.39	47.14 ± 2.23	63.30 ± 4.47
GMT	79.08 ± 0.66	75.79 ± 1.18	75.23 ± 1.25	74.08 ± 1.11	79.41 ± 0.78	47.72 ± 1.41	67.38 ± 2.04
gPool	76.69 ± 1.48	72.57 ± 1.51	72.83 ± 0.90	67.02 ± 2.25	76.35 ± 2.67	47.00 ± 2.33	65.59 ± 2.73
ASAP	78.94 ± 2.28	76.11 ± 2.39	73.48 ± 0.42	70.07 ± 0.55	78.50 ± 0.94	46.91 ± 0.54	68.50 ± 1.96
TAPool	80.65 ± 2.54	77.23 ± 2.98	76.50 ± 3.46	73.11 ± 2.54	75.60 ± 1.79	**49.95 ± 2.44**	71.45 ± 3.28
EdgePool	79.54 ± 1.96	77.20 ± 1.51	78.88 ± 0.68	76.10 ± 0.55	79.36 ± 0.71	47.21 ± 0.61	70.20 ± 1.34
SparsePool	**81.97 ± 2.22**	**78.07 ± 1.04**	**79.89 ± 0.73**	**77.24 ± 1.78**	**80.30 ± 0.68**	49.25 ± 0.90	**72.53 ± 1.84**

**Table 3:** Classification accuracy (%) of SparsePool with different GNN backbones (GCN vs. GAT) on test sets. Best results are in bold.

Dataset	D&D	Protein	NCI1	NCI109	COLLAB
TAPool	80.65 ± 2.54	77.23 ± 2.98	76.50 ± 3.46	73.11 ± 2.54	75.60 ± 1.79
EdgePool	79.54 ± 1.96	77.20 ± 1.51	78.88 ± 0.68	76.10 ± 0.55	79.36 ± 0.71
SparsePool (GCN)	81.97 ± 2.22	**78.07 ± 1.04**	79.89 ± 0.73	77.24 ± 1.78	80.30 ± 0.68
SparsePool (GAT)	**82.01 ± 1.04**	77.68 ± 1.04	**80.89 ± 0.73**	**77.53 ± 0.60**	**81.08 ± 1.54**

## Data Availability

The data presented in this study are openly available in the TUDataset repository at https://chrsmrrs.github.io/datasets/ (accessed on 1 March 2026) [43]. These data were derived from resource available in the public domain.
